# Exploring the bases for a mixed reality stroke rehabilitation system, Part I: A unified approach for representing action, quantitative evaluation, and interactive feedback

**DOI:** 10.1186/1743-0003-8-51

**Published:** 2011-08-30

**Authors:** Nicole Lehrer, Suneth Attygalle, Steven L Wolf, Thanassis Rikakis

**Affiliations:** 1 School of Arts, Media and Engineering, Arizona State University, Tempe, USA; 2 Department of Bioengineering, Arizona State University, Tempe, USA; 3 Department of Rehabilitation Medicine, Emory University, Atlanta, USA

## Abstract

**Background:**

Although principles based in motor learning, rehabilitation, and human-computer interfaces can guide the design of effective interactive systems for rehabilitation, a unified approach that connects these key principles into an integrated design, and can form a methodology that can be generalized to interactive stroke rehabilitation, is presently unavailable.

**Results:**

This paper integrates phenomenological approaches to interaction and embodied knowledge with rehabilitation practices and theories to achieve the basis for a methodology that can support effective adaptive, interactive rehabilitation. Our resulting methodology provides guidelines for the development of an action representation, quantification of action, and the design of interactive feedback. As Part I of a two-part series, this paper presents key principles of the unified approach. Part II then describes the application of this approach within the implementation of the Adaptive Mixed Reality Rehabilitation (AMRR) system for stroke rehabilitation.

**Conclusions:**

The accompanying principles for composing novel mixed reality environments for stroke rehabilitation can advance the design and implementation of effective mixed reality systems for the clinical setting, and ultimately be adapted for home-based application. They furthermore can be applied to other rehabilitation needs beyond stroke.

## Background

Approaches to rehabilitation training grounded in motor learning can increase the opportunity for restitution of function following stroke [1]. Principles in motor learning can inform the design of rehabilitation therapies by establishing guidelines for practice and types of feedback to use. A review of motor learning studies indicates that distributed practice with variability leads to better retention of skilled actions [1-3]. Specificity and repetition of exercise within rehabilitation training can also be effective in promoting motor learning within unassisted, goal-directed practice [4].

Virtual and mixed reality environments have been developed to provide effective mediums that utilize motor learning principles for rehabilitation training. Virtual environments tend to immerse the participant within a completely simulated space, while mixed reality environments integrate both digital and physical elements. Because mixed realities provide interactive experiences that are situated in physical reality, such environments have the potential to provide mediated training that still facilitates generalization and transference of knowledge from therapy to activities beyond rehabilitation [5]. The application of motion sensing technology, such as optical motion capture systems, towards rehabilitation practice can provide highly accurate information describing patient performance. Linking motion-sensing technology with visual and audio feedback can create engaging, interactive experiences that provide detailed information on performance for the stroke survivor in a manner that facilitates active engagement and sensorimotor learning. The use of augmented feedback to engage the user in repetitive task training can also be effective in reducing motor impairment [6]. Several groups have explored the application of motion capture based virtual reality within upper extremity rehabilitation [7-14], though the extent to which training with augmented or virtual realities is more effective than traditional therapy techniques is still under investigation.

Principles based in motor learning, rehabilitation, and human-computer interaction, among other disciplines, can guide the design of effective interactive systems for rehabilitation. An interactive system should provide integrated training of movement aspects related to the impaired task. The division of a task into subcomponents and practice of these subcomponents do not necessarily facilitate learning of the entire action, unless the integrated action is also practiced [1]. A variety of feedback scenarios can be implemented for interactive systems, yet not all feedback scenarios are appropriate to communicate information on integrated movement performance. Some feedback can even be detrimental if it fosters too much user dependence or concern with performance during movement [2]. Excessive, explicit information about the task can interfere with implicit learning by the stroke survivor [15]. However, rehabilitation systems that encourage independent detection and understanding of performance errors can facilitate learning of the motor task [16]. The amount of feedback should be controlled for and optimized to each individual stroke survivor's progress throughout therapy [11,17]. Finally, each component of training should be adaptable to appropriately challenge the stroke survivor as therapy progresses.

Although the above principles are by now well established individually, there is still a lack of a unified approach that connects these key components into an integrated design and can form a basis for a generalizable interactive rehabilitation methodology for stroke. This paper proposes basic principles for such an integrated design as they were applied to the creation of an Adaptive Mixed Reality Rehabilitation (AMRR) system for a reach and grasp action. Preliminary data from a study employing the AMRR system has demonstrated the system's ability to facilitate integrated recovery and is included within the companion paper.

**Figure 1:**
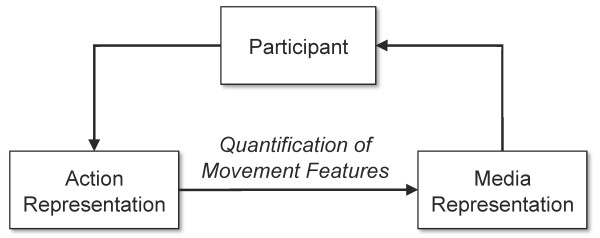
**Overview of an integrated approach to designing mixed reality rehabilitation systems**. An action representation is developed and quantified. Quantification allows for the action representation to be communicated through a media representation to the participant for engagement and intuitive communication of performance to facilitate self-assessment.

Because an established methodology for interactive stroke rehabilitation does not yet exist, we derive some of our key principles from interactive learning and general motor rehabilitation theories. Our resulting methodology provides guidelines for the development of an action representation, quantification of action, and the design of interactive feedback (Figure 1). We first present the underlying methods for creating an action representation by way of integrating phenomenological approaches to interactive systems and rehabilitation principles. We then present our resulting action representation for a reach and grasp, general methods for quantification, and compositional principles for designing interactive media-based feedback.

## Methods

### Development of an Action Representation

**Figure 2:**
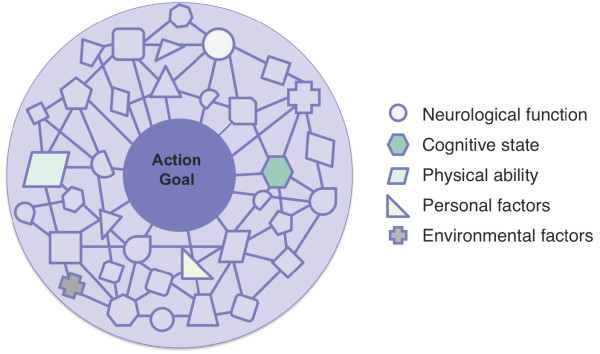
**Conceptual representation of all elements comprising the action space network**. The large central node of the action space network is the action goal. Surrounding nodes contribute to the action goal in varying degrees, and also influence each other within the network. Uniquely shaped nodes represent different contributing parameters of the network.

An overwhelming number of parameters and influences, such as neurological function, cognitive state [17], and physical ability [18], affect the performance of an activity. The full set of parameters or influences affecting an individual's performance of an activity compose an action space, which is considered to have a network structure (Figure 2). Parameters delineating the space do not act in isolation but contribute to an interconnected system of influences affecting performance and achievement of the action goal.

Due to its high complexity, identifying and measuring all parameters of an action space are not possible. The design of an interactive rehabilitation system requires an action representation that simplifies the full action space into fewer parameters that are representative of the entire action. This simplified representation focuses on the key elements of movement being trained and their interrelations. Such a representation provides a manageable number of parameters to monitor in real-time, quantify, and communicate through feedback. An action representation also facilitates common understanding of the movement among experts from different fields. This representation should therefore address the needs of the clinician and skills of a computational expert to facilitate the provision of a common basis for designing the interactive system. We propose that embodied interaction principles arising from phenomenology offer a well-informed starting point for the development of simplified representations of actions for interactive rehabilitation. These phenomenology principles must be integrated with relevant motor learning and rehabilitation principles.

### A Phenomenological Approach to Action and Interactive Computing

Principles derived from phenomenology can facilitate understanding of embodied interaction and the development of interactive interfaces. Embodied interaction stresses the importance of knowledge gained through the body's experience interacting with its environment [19]. Although embodied knowledge arises from simple everyday activities, the process of obtaining embodied knowledge is not a simple phenomenon. As depicted in Figure 2, an action goal is accomplished within the context of a highly complex network of factors and influences generated by the relationship between the user and his or her environment.

Interactive learning is managed by the continuous process of coupling, separation, and re-engagement among the body, an external tool, and an action [19]. Focus on completing the action goal (such as browsing a webpage for specific content) allows for coupling the tool (a computer mouse) and the action (moving the mouse while searching the webpage). Coupling means that the activity is being undertaken without conscious awareness of how the body is using the tool to accomplish the action goal. Failure to achieve the action goal causes decoupling (browsing the webpage and using the mouse become separate components), which allows for exploration of performance components towards achieving the action goal (contemplation of how to better orient the mouse so that it functions properly again). Finally, re-engagement is the re-coupling of tool and action that allows for renewed focus on achieving the action goal [19]. Tool/action coupling is also mirrored in the theory of embodied cognition [20], as interaction with the environment through one's body is in fact how one perceives the environment. In this case, the body is the tool that is accomplishing the action.

Phenomenology-based approaches to understanding embodied knowledge through coupling among action, body, and tool are highly relevant to systems focusing on functional recovery. During rehabilitation, the action goal, activity, and body function need to be considered together and separately under different circumstances. Phenomenology also maintains that knowledge of one's environment and body arises through accomplishing everyday activities [21]. This approach parallels the concept of repetitive task training for rehabilitation [22] by allowing for the breaking down of daily activity into a series of goal-oriented actions repeated throughout the day in various forms. Furthermore, completion of multiple action goals with different degrees of similarity contributes to the accumulation of embodied knowledge.

Current motor learning theory also aligns with basic concepts relevant to phenomenology and embodied cognition. Motor control is considered to be a modular process, in which the goal and action plan precede execution without consideration of limb dynamics in the initial stage of planning. During execution, body dynamics are continuously adapted to realize the activity plan and achieve the action goal [1]. The motor system is designed with action as its core, rather than movement alone [23].

**Figure 3:**
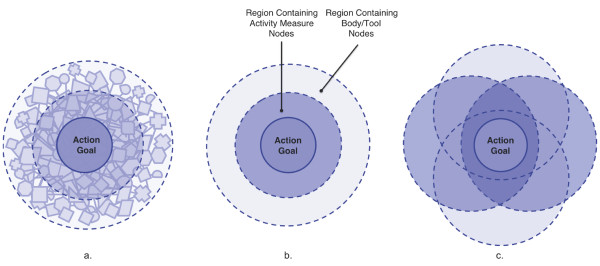
**Simplified conceptual representation of the action space network**. The action goal is nested as the central focus of the action, to which other nodes of the network contribute (3a). The visual presentation is simplified by showing only relationships between the goal and two overarching categories of nodes. When focus is on the action goal there is full coupling of categories, shown by full overlap of categories (3b). Decoupling for exploring body/tool function relationships to action is shown by breaking down categories and reducing overlap (3c).

Phenomenological constructs support a representation of action as a nested network representation depicted in Figure 3a: the action goal is nested as the central focus of the action, to which other nodes of the network contribute. Because the action space network is a highly complex space, the visual presentation is simplified in Figure 3b by showing only relationships among the goal and two overarching categories of nodes (body/tool and activity measure categories), rather than attempting to show relationships among individual elements. The realization of the action goal is at the center of the network, and overlaps strongly with continuous activity measures (e.g., actively searching webpage content with a mouse). Both the action goal and the activity measures overlap with body/tool functions (e.g., how is the mouse being grasped) that influence the activity and the completion of the goal. Hierarchy with respect to accomplishing the action goal can be demonstrated by distance to center (important categories are more centralized) and relationship can be depicted through overlap (interrelated categories have higher overlap). When focus is on the action goal, there is full coupling of categories, shown by full overlap of categories in Figure 3b. Decoupling for exploring body/tool function relationships to action is shown by breaking down categories and reducing overlap, as shown in Figure 3c.

### A Rehabilitation Approach towards Understanding Action

Learning through repetitive action, and consideration of activity and body function measures in the context of achieving action goals are also focal parts of current rehabilitation approaches. However, the full-scale integration and seamless coupling and decoupling of all action, body, and tool elements present in the action network of non-impaired subjects (Figure 3b-c) cannot totally transfer to rehabilitation training.

Recent publications [24,25] support the necessity to simplify aspects of the action space being monitored or attended to within rehabilitation. In this context, understanding action is achieved by directed efforts on only a few quantifiable components, and allowing for varied levels of coupling among goal, activity, and body function components based upon stroke survivors' abilities.

**Figure 4:**
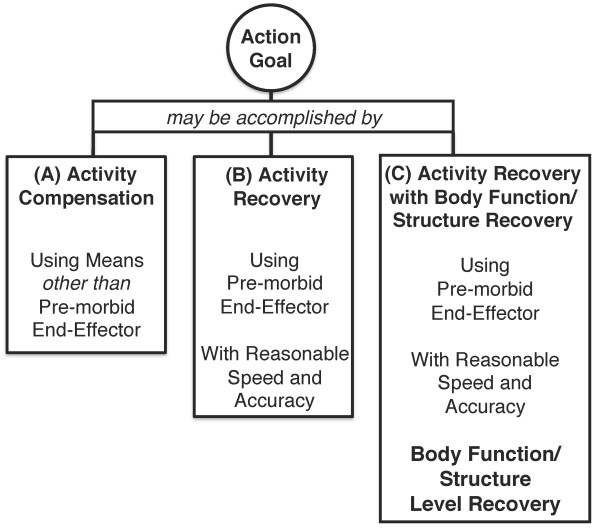
**Rehabilitation Approach to action for discriminating behavioral recovery and compensation, adapted from **[24] . Three types of action leading to goal accomplishment include activity compensation, activity recovery, and activity recovery with body function/structure recovery.

Levin, Kleim, and Wolf have proposed a classification system for discrimination between recovery and compensation in patients following stroke within the context of the World Health Organization International Classification of Functioning (ICF) model [24]. They identify three kinds of goal accomplishment in stroke rehabilitation. Activity compensation describes goal accomplishment by means of an alternative end-effector with no time or accuracy constraints. Activity recovery describes goal accomplishment by the pre-morbid dominant end effector with reasonable speed and accuracy, without body function compensation constraints. Finally, activity recovery with body function/structure recovery describes usage of the pre-morbid end effector with reasonable speed and accuracy, without significant body compensation. In this case, the participant's use of the end effector and task-related body components are within the range of efficient unimpaired performance. Figure 4 displays a graphic representation synthesized from the Levin, Kleim and Wolf approach.

Kwakkel takes a related approach and notes that rehabilitation therapies should not seek to achieve full restitution, irrespective of patient capability [25]. Rather rehabilitation therapies must be adjustable and adaptable to fit the patient's prognosis for recovery and progress during therapy without increasing patient frustration. Understanding the balance between restitution of body functions and compensatory behavior is crucial for designing therapies that are well suited for the patient at his or her particular stage of recovery [25]. Thus, the action space representation for rehabilitation cannot assume a full, continuous, and integrated calibration of activity and body function aspects of the action network as within the phenomenological model (Figure 3).

The need for quantification further promotes a simplified action representation. Recent publications [11,17] discuss the pressing need for quantitative evaluation of customizable approaches to stroke rehabilitation. Interactive rehabilitation also demands detailed, quantitative, real-time evaluation to reveal to the participant the state of his action network. Most stroke survivors require assistance in reconnecting (or forming new connections between) goal accomplishment and their action network. Despite current advances in theory, methodology, and computation, tracking and revealing the state of the full action network of an impaired user is very challenging. Even if one could measure and replicate the full action space, offering real-time assessment and feedback on all parameters would produce an enormously complex experience that neither therapist nor patient could parse and utilize in real-time. Focusing on a few key components that adequately measure activity and body function in the context of goal accomplishment is necessary.

## Results and Discussion

An integration of the above approaches can help us define the key characteristics for a simplified representation of action for effective interactive stroke rehabilitation. Considering in parallel the full set of influences of the WHO IFC model (including neurological and cognitive influences, as well as the broad internal and environmental factors of a stroke survivor's life) is too complex a goal. The first focus should be the stroke survivor's own action space with emphasis on the physical manifestation of his actions. The overall representation of this limited definition of action space should maintain the nested network form of a non-impaired action network, with the action goal as the center. However the representation should include only a few key movement components that are integral to efficient goal accomplishment and can be monitored, calculated, and communicated in real-time. The overall organization of these components should follow the activity/body function categorization. Within these overarching categories, sub-categories should be structured that are commonly used in rehabilitation and facilitate handling of components in real-time through groups pertinent to the action (e.g., targeting, joint function). Strength of coupling among different components and subcategories should be shown only at a general level, as specific correlations will vary for different patients at different levels of recovery.

Selection of the kinematic components and sub-categories that populate the simplified action representation should be derived from motor control principles and relevant rehabilitation literature and practice. For reach and grasp actions as an example, the brain is thought to control movement by considering the end-point as the guiding reference [26,27]. The underlying theory of common coding [28] supports the premise that action plans are anchored by elements that can provide common representations of action and perception. In reach and grasp movements, the end-point, as the major interactor with the environment and the action goal, becomes the common planning anchor. Reaching trajectories involving multiple joints consistently have nearly invariant kinematic characteristics, such as straight-line trajectory paths and bell-shaped velocity profiles [1,29,30] that are derived from end-point activity and are strongly correlated to efficient accomplishment of the activity goal. Thus, the representation of the reach and grasp action focuses significantly on the end-point, monitored continuously over time and space. Key kinematic features required to monitor, evaluate and communicate the participant's reaching performance are extracted from the end-point movement alone. Within the action representation, goal accomplishment is shown to be strongly affected by the activity sub-categories that are populated by kinematic components extracted from end-point data.

In a reach and grasp action representation, parameters within the body function category should focus on measurements of body function issues affecting a large majority of stroke survivors. The appropriate timing and execution of forearm rotation in the context of a reach and grasp action can pose challenge for stroke survivors [31] and may require monitoring and feedback for assistance. Elbow extension is an aspect of movement by hemiparetic individuals that often requires encouragement to achieve a maximum reach. The lack of elbow extension can result in compensatory movements using the shoulder and trunk. Stroke survivors increase their use of shoulder and torso body structures to compensate for deficiencies in the range of motion of their distal joints [32]. Even if multiple compensatory strategies are used, the stroke survivor may still be able to successfully move the end-point to a target [32] with a seemingly correct pattern. Thus, individually monitoring more proximal components, such as shoulder and torso movements, is necessary. Monitoring elbow lift in the vertical direction prior to reach initiation can detect preemptive shoulder compensation associated with movement initiation [33]. Measured joint angles offer information about the range of movement of individual joints during the reach, while measuring inter-joint correlations can reveal relationships among different joints. These key aspects of body function that may influence a stroke survivor's reaching movement should therefore be incorporated into the action representation.

### Representing Reach and Grasp Action as a Nested Network of Functional Features

**Figure 5:**
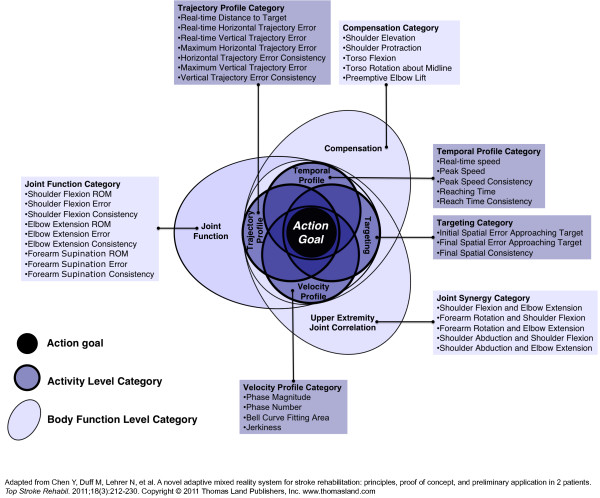
**Action Representation for a Reach and Grasp**. Kinematic parameters are listed within seven sub-categories: Four activity level sub-categories (dark background) and three body function level sub-categories (light background). Overlap between categories indicates the general amount of correlation among kinematic parameters with respect to action goal completion. Categories located close to the center of the representation are higher in the hierarchy of training goals, with greater influence on goal completion.

Figure 5 presents an example of a simplified action representation for stroke rehabilitation, which represents the reach and grasp action as a nested network of key kinematic parameters. These kinematic features are organized into seven sub-categories of movement attributes, based upon operational similarities within the reach and grasp movement. The seven sub-categories are classified as either activity or body function measurements.

*Temporal Profile, Targeting, Trajectory Profile*, and *Velocity Profile *are the four activity level sub-categories that contain kinematic features derived from the end-point activity (movement of the hand over space and time). Kinematic features within the four activity level sub-categories are highly correlated in terms of activity level training and have the greatest influence on the efficient completion of the action goal. Therefore, these four sub-categories significantly overlap and are located close to the center of the representation.

The remaining three sub-categories, *Compensation, Joint Function*, and *Upper Extremity Joint Correlation*, are body function level sub-categories, which include kinematic parameters that, once recovered, reflect pre-morbid movement patterns of specific body structures. Behavioral recovery of specific body structures, such as elbow extension or wrist rotation, may certainly influence achievement of the action goal. However, the recovery of pre-morbid body structure movement patterns is not required for action completion, and may in fact be undesirable as a training focus for some stroke survivors. Thus, the three body function level sub-categories are located on the outer edges of the representation and may be focused upon at the discretion of the clinician. Parameters within these sub-categories may be focused upon in a less correlated manner in training than activity level sub-categories, and thus are visually depicted with less overlap. Because much of the high-level behavior during reaching and grasping can be understood from the end-point behavior, the action representation shown in Figure 5 does not include the monitoring of fingers and grasping as a continuous measure. However this representation may be modified to include grasping as an additional body function category.

The *Action Goal *node shown at the center of the representation in Figure 5 is not considered a separate sub-category but rather a composite node integrating aspects of the surrounding sub-categories. Because the action space network is a highly complex space, the action representation does not attempt to show relationships among individual kinematic components, only relationships among sub-categories and overarching activity and body function level categories. Two key relationships among kinematic features emerge from the action representation: hierarchy of training goals and general correlation. The correlation shown is only general (and indicative), as the specific relationships among individual kinematic parameters will vary for each stroke survivor. The resulting representation can form the basis for quantifiable, adaptive, manageable re-learning of the relationships among action goal, activity and body functions within interactive stroke rehabilitation. For this abstraction of movement to be used by clinicians to evaluate patients and by the media expert to design feedback, the kinematic components must be quantified in a manner that reflects how they can be implemented during treatment.

### Quantification of the Stroke Survivor's Movement

An action representation simplifies the understanding and monitoring of the action space but does not provide information on how to evaluate the attributes within the representation. In reach and grasp actions, for example, velocity profile has been identified as an important feature within stroke rehabilitation literature [1,29,30] and is thus an activity level sub-category of our representation. However, meaningful assessment of the velocity profile cannot occur without specific measurable parameters that accurately define and evaluate this aspect of the movement. A velocity profile can be characterized by its peak magnitude, or described as an overall shape compared to an idealized bell-shaped curve. The velocity profile of each reach can be considered separately, or emphasis can be given on consistency across profiles of multiple reaches. Thus, establishing quantification of these features enables a precise definition of how velocity profile is being assessed. A simple, quantified representation of action can facilitate online and offline assessment of the movement by the clinician and form the basis for the production of feedback that enables self-assessment by the stroke survivor.

#### Detailed assessment required for feedback generation

Many clinical outcome measures, such as those accessing neurological deficit, ability to perform tasks, and quality of life [34] have been developed to evaluate recovery or disability post stroke. Although currently available quantitative clinical scales are imbued with consistent and reliable protocols, each clinician can approach these measures uniquely. A review on the clinical interpretation of stroke scales emphasizes that without awareness of the advantages and limitations associated with each measure, the potential exists for inconsistent selection, application, and evaluation among practitioners using these available outcome measures [34]. Use of these existing scales therefore cannot guarantee detailed, standardized measurements of the kinematic features within the action representation.

Furthermore, currently available quantitative scales cannot easily capture real-time, high-resolution information on movement that is necessary for detailed assessment of each movement component and the digital generation of real-time continuous feedback. Clinicians using existing measures may consider the overall performance of a movement, or of an individual feature of movement, across repeated actions (i.e., reaches). However, monitoring multiple aspects of movement and their interrelationships at a high level of detail is very difficult. Clinician observations and assessments are often available as post-movement annotations and cannot provide a quantified value in terms of how each individual activity or body function component affected the overall performance score. The ability to produce such relevant information in a timely fashion is important for assessing and providing feedback on the entire action, as opposed to segmenting assessment on the performance for only one body structure at a time. Detailed aspects of movement that should be communicated to the stroke survivor, including magnitude and direction of error for each component, are not possible without the level of detail obtained by quantifying the action by motion capture or other means. Motion capture and computational analysis can offer detailed kinematic information on multiple aspects of movement in real-time. Quantification of movement by means of computational assistance and archiving allows for the documentation of movement performance that can be accessed and analyzed during and after a single set, or after multiple sets in order to convey performance consistency measures.

#### Application in practice

Finally, one must determine how to map computational analysis of kinematics to meaningful assessment scales. We propose that each kinematic attribute should be given a non-impaired performance range. This non-impaired performance range should be determined from kinematic data derived from a sample of unimpaired subjects performing multiple repetitions of the relevant task (i.e., multiple reaching and grasping tasks). For each kinematic attribute, performance data should also be collected from stroke survivors possessing a wide range of impairment, spanning between minimal and maximal impairment for that movement attribute. A model should then be constructed from the collected performance data of both unimpaired participants and from stroke survivors by mathematically fitting these data to a continuous function. This function may be used to place raw values from computational analysis on a normalized scale (ranging between 0 and 1) to determine the amount of impairment for that kinematic attribute. Processes should also be developed for integrating measurements of individual kinematic attributes into measurements of sub-categories, and overall measurements of the full movement. An example of such a standardized measure for reach to grasp movement [35] has been developed, based on the kinematic features of the action representation, and in the future may be expanded to include muscle activity measures as well. In the context of the reach and grasp representation, quantified assessment relies on using three types of reference data for comparison of stroke survivor performance to unimpaired movements: trajectory references, joint angle references, and torso/shoulder movement references. Each profile is scaled to patient-specific values and as a function of the normalized distance from the hand to the target [36].

Working with practitioners to determine how these computationally derived functions correlate with the clinician's assessment is a necessity. The functions must be tested with stroke survivors possessing differing degrees of impairment, and then adjusted so as to better fit the experienced clinician's rating. This method of iterative design research is crucial to the development of quantitative evaluations that are meaningful to clinical practice. These quantitative measurements can then form the basis of the feedback that the stroke survivor experiences as a result of his movement.

### Composing Media-Based Feedback

Interactive media-based feedback can provide engagement, intuitively communicate performance, and facilitate self-assessment by the stroke survivor. Multimedia compositions, such as films, can provide an external source of encouragement and engagement. Interactive multimodal media systems (combining audio, visual and tangible elements) have been used extensively to facilitate active learning in general [37-41], and motor learning specifically [9-11,42]. However, little evidence exists regarding the standardized application of media composition and interactive learning approaches to stroke therapy for enhancing rehabilitation outcomes. In the following section we present four principles that may guide the creation of effective feedback for mediated motor learning for stroke rehabilitation: abstract representation, feature selection, form integration and coherence, and adaptive design.

#### Abstract representation for recontextualization, active participation, and generalization

When providing media-based feedback on performance, selection of the appropriate media content can be extremely influential on how the task is perceived and performed by the participant. Abstract representation can provide feedback that does not directly represent the training task but is tightly coupled to and directly controlled by a participant's action. The ability of abstract representation to encourage recontextualization, active participation, and generalization support that its provision as feedback may be highly conducive to mediated motor learning.

Recontextualization facilitates a new perspective or understanding towards a learning scenario by changing the context of an existing challenge [43]. Recontextualization of the training task using abstract representation may assist a stroke survivor to discontinue reliance upon pre-existing inefficient, and possibly detrimental, movement strategies used in post-stroke daily living that prevent the opportunity for restitution [44]. Virtual reality environments that directly represent a training task may reiterate existing frustrations associated with the task's difficulty by not supporting recontextualization. Presenting a virtual scene that depicts an arm grasping a cup, for example, may evoke memories of past failed attempts and consequences [45] that can negatively affect performance. Furthermore, virtual environments that attempt to realistically depict human forms may introduce undesirable artifacts that distract the viewer [46]. The use of abstract feedback can encourage active participation and problem solving by requiring the participant to determine the causality between his action and the corresponding change in feedback. For example, within the AMRR system, completing a reaching task controls the performance of a media-based task of forming an image (presented on an LCD screen) and creating a musical progression (heard through speakers). The metaphorical reconstruction between action and feedback requires active engagement, which supports parallel cognitive and motor learning by the stroke survivor [17]. Encouraging problem solving during therapy has been demonstrated to be a key contributor to promoting neural plasticity for rehabilitation [47,48].

Effective feedback for motor learning that encourages active participation generally should not be prescriptive, or directly instruct how to solve a problem. For example, prescriptive feedback might provide an explicit trajectory path for a hand to follow during a reaching task. Though some forms of prescriptive feedback have been identified as effective for novice learners [16], if consistently applied this approach may create dependencies on the feedback and therefore is less likely to promote active, independent learning [49,50]. Some types of feedback may have unwanted prescriptive effects on performance. A prominent, regular rhythmic pattern may encourage the stroke survivor to attempt to move in the rhythm of the music rather than develop his own efficient, timing pattern for the task [51,52]. Familiar musical songs have one fixed ideal form, the form with which the user is familiar [53]. Such songs cannot be used in an adaptive manner during therapy and may shift the focus of the stroke survivor to the performance of the feedback (aiming for the ideal form) rather than the performance of the movement itself. For example, the participant may move faster to achieve a faster musical speed because he/she does not favor the selected play back speed caused by his correct movement speed. Even when such artifacts do not arise, interactions with fixed-form feedback can only communicate to the user the amount of error in terms of distance from the "ideal" and gross direction for improvement (e.g., move faster) but cannot communicate detailed aspects for improvement within the context of the overall action (e.g., shape of acceleration/deceleration). Similar challenges arise when using representational mappings that do not reflect the desired form of the action (e.g., mapping a reach and grasp action to a tennis swing within a Wii game).

Abstract, novel (non-familiar) feedback focuses the participant's attention on the form of the action, and can thus emphasize deviation from efficient performance, and provide intuitive, detailed direction for improvement. For example, within the AMRR system, linearity of a reaching trajectory is encouraged by stretching components of an animated image in the direction of hand deviation, to intuitively signal movement in the opposite direction of the stretch in order to reduce the distortion. During the performance of the trajectory, the image is broken into many small components and the user interacts with the movement of the abstract animated components. The image that is animated may be familiar, but the user only sees the image at the beginning of the action (establishing a familiar goal) and at the end (rewarding a successful reach).

Because abstract media-based feedback does not reflect a specific situation grounded in physical reality, it can be applied to several different training scenarios. Application across scenarios promotes generalizeable learning by communicating the invariant aspects of movement across different types of tasks. For example, mapping of hand speed to rhythm can be applied whether the user is reaching towards a target across his midline or within a sagittal plane. Braun et al [54] demonstrated that when subjects are exposed to varying tasks of the same structure, motor control processes could extract the structure of the task, suggesting that the human motor control system relies on structural, generalizeable learning for skill acquisition.

#### Feature spaces for designing media feedback

When composing media-based feedback for stroke rehabilitation, the selection of appropriate feedback features is critical to the successful communication of movement performance. Given that an action representation lists multiple aspects of movement and their general relationships with respect to achieving the action goal, we propose that a multidimensional feature space is necessary to appropriately design media that can communicate both individual and integrated aspects of movement.

While the categorical division of feedback into knowledge of results (KR) versus knowledge of performance (KP) has become one accepted paradigm for discriminating different types of feedback for motor learning, we propose a more nuanced feature space with multiple dimensions that allows for the development of feedback within mixed reality systems for stroke. High resolution sensing technologies applied within interactive rehabilitation systems support far more detailed media feedback at multiple timescales than was previously available, introducing new types of feedback that relate to both KR and KP. Furthermore, differences arise among definitions of KR and KP in terms of both type of information conveyed and time of delivery with respect to movement. KR has been defined as information provided on goal outcome, while KP has been defined as information on movement quality [2,55]. Recent publications also acknowledge KR as feedback provided on the outcome of skill performance [16] in addition to goal achievement. In terms of delivery, while some literature define KR and KP as feedback provided after movement is complete [2,55] others describe KP as feedback that may also be provided simultaneously to movement [16,17].

**Figure 6:**
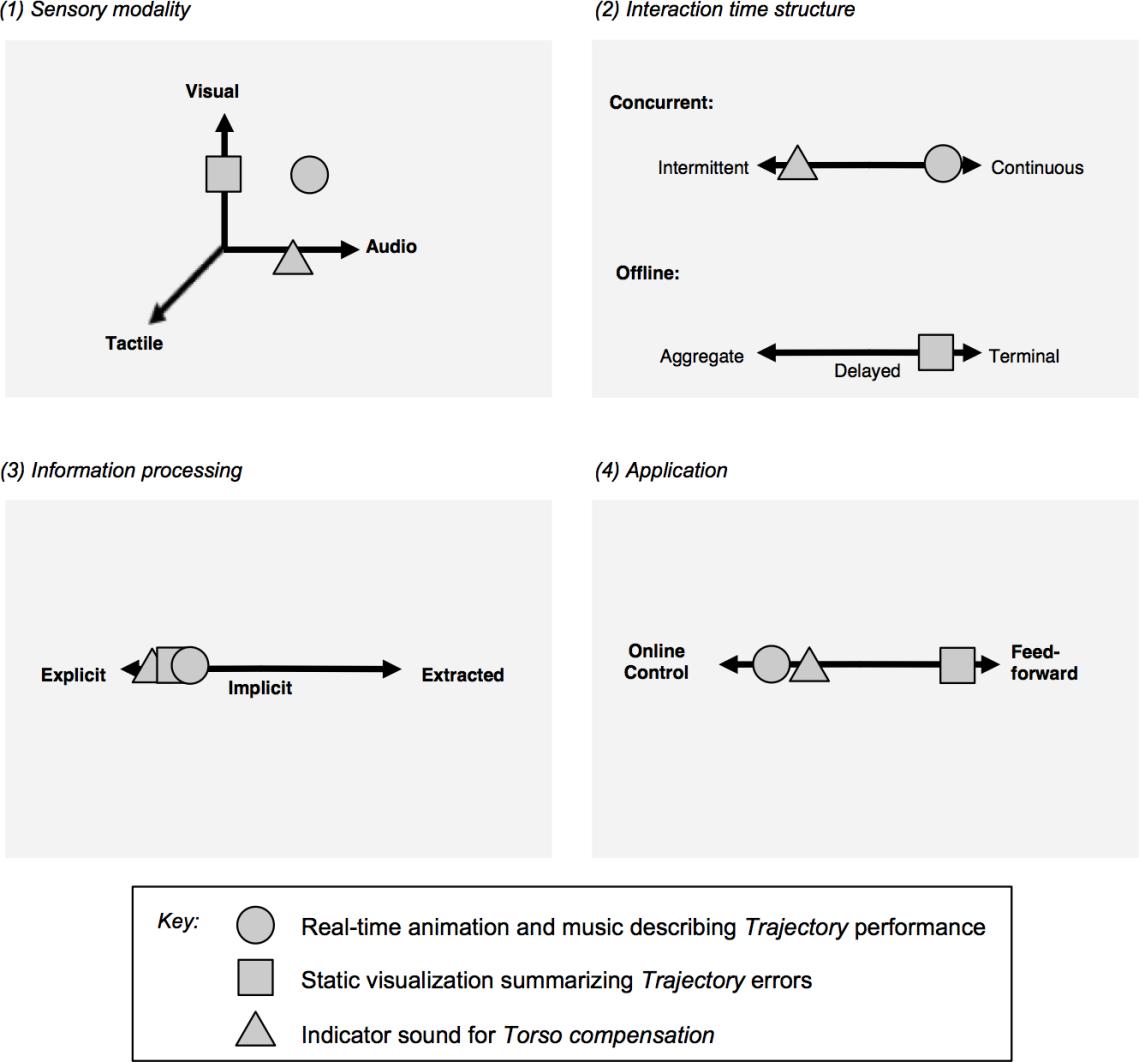
**Four feature spaces categorizing feedback for mediated motor learning, provided with example feedback mappings for reaching trajectory and torso compensation from the AMRR system**. The example feedback mappings for trajectory and torso compensation are characterized by the location of three unique points placed within each feature space. See descriptions of each feature space in section titled ***"***Feature spaces for designing media feedback".

Therefore we have identified four feature spaces to address the multiple subspaces of both KP and KR for consideration when designing media-based feedback for stroke rehabilitation: sensory modality, information processing, interaction time structure, and application. Each feedback element communicating a movement component therefore has four sets of coordinates (one for each space). Figure 6 shows the example coordinates for feedback components assigned to trajectory error and torso compensation within the Adaptive Mixed Reality Rehabilitation (AMRR) system. In the following sections we describe each feature space and provide guidelines for its usage in developing effective movement-feedback mappings.

##### Sensory Modality

Sensory modality appropriateness [56] refers to the extent to which a specific sensory modality provides the most accurate or appropriate sensory information [57]. *Visual *feedback is best suited for communicating spatial information, such as providing guidance for correcting trajectory errors in goal-directed arm movement [58]. The use of visual perspective is also an intuitive communicator of spatial depth [59,60] and visual point of view [61]. For example, in a visualization for a directed reach and grasp task, visual perspective can help indicate distance between the hand and target, as well as the observer's position relative to the target. *Audio *feedback is best suited for communicating temporal knowledge [62]. Movement patterns requiring complex timing or synchronization can be trained effectively through musical rhythm [52,63,64]. A study conducted by Thaut et al demonstrated the ability of auditory rhythm to effectively entrain motor patterns in stroke rehabilitation [65]. Tonal theory suggests that the use of chord sequences and melodic contour (change in pitch over time) can impart a sense of forward movement [66-69] and can be used to encourage, monitor, and time a progression towards the completion of the action goal [70]. *Tactile *feedback is utilized by the haptic system to confirm target acquisition [71] and modulate grip force for stable grasping [72]. Tactile feedback is also used to detect when contact is made or broken with surfaces in the environment, which can be applied for anticipatory control based on memory from previous interactions [69] and can provide guidance during a supported (target located on a table) reaching task. The Mixed Reality Rehabilitation group at ASU has conducted a study in which these audiovisual communication principles were successfully tested in interactive rehabilitation for five patients with hemiparesis secondary to stroke [73].

##### Information Processing

Depending upon the type of movement parameter being communicated, feedback should promote the appropriate type of information processing. Here we define the information processing space as a continuum ranging from explicit, to implicit, to extracted. Feedback that promotes *explicit *information processing is one in which the relationship between causal action and feedback is direct and readily apparent, without contemplation and upon limited interaction. An example of feedback promoting *explicit *information processing within the AMRR system is the animated movement of an image to the right presented to the participant as his hand moves towards the right during a reaching task. While the example of trajectory feedback communicates an overt indication of error upon limited interaction, information encoded within feedback promoting *implicit *information processing does not. Feedback that promotes an *implicit *process requires multiple interactions by the participant, and may be understood through exploration and resulting self-discovery. For example, within the AMRR system, an acceleration/deceleration pattern of notes can be derived when speed of movement is mapped to speed of musical rhythm, but requires multiple interactions before the participant can implicitly utilize the resulting rhythmic shape to achieve a bell-shaped velocity. Multiple feedback components that are designed to be integrative through their similarity or promote interrelationships through their dissimilarity can support *extracted *information processing for more complex aspects of movement that are multidimensional in nature, such as the velocity profile of the hand while reaching. The *extraction *process is performed by the participant through his experience with the interactive feedback and may involve either the combination of multiple parallel feedback mappings (e.g., the concept of velocity as space/time, communicated through an integration of visual and music progressions); or the deduction of causalities between different feedback indicators (e.g., the relationship of shoulder compensation feedback to trajectory error feedback). This relationship requires the most prolonged exposure with the feedback environment of an interactive system in order to support the forming of an extracted mental construct by the participant. Variation in the degree of problem solving required by different types of feedback can provide an experience that is balanced between encouragement and self-discovery to optimize learning [2,74]. Feedback requiring multiple processes may encourage both explicit and implicit learning, each of which are often present in most learning scenarios with varying amounts of contribution from each [74].

##### Interaction Time Structure

The structured delivery of feedback over time should be based on the movement component to be communicated. *Concurrent *feedback [16,17] is given instantaneously, in real-time or exhibiting no perceptible delay, with respect to the participant performing an action. Aspects of movement that are continuously monitored by the mover while performing an action may require *continuously *delivered *concurrent *feedback for detailed knowledge to correct error. For example, a reach and grasp action requires continuous feedback on end-point spatial progress towards a target [58]. Types of feedback that are both *concurrent *and *continuous *may be referred to as (media) streams, which describe the continuous flow of media information that is provided in immediate response to the participant's movement.

Aspects of movement irregularly relevant to performance of a specific action [75], such as torso compensation during a reaching activity, are more appropriately described by *intermittent *feedback so as to not interfere with the continuous monitoring of the end effector. Intermittent feedback in this context is *concurrent *feedback that is both limited in the amount of information given, such as on/off feedback, and is provided only when relevant to performance (e.g., a brief audio indicator provided when torso compensation occurs). Audio media is often the most appropriate medium for intermittent indicator feedback, as it does not distract from continuous visual monitoring of the end-effector but can connect the temporal placement of real-time events, such as real-time error correction, within overall memory of continuous aspects of action.

Feedback that is provided *offline *can be *terminal*, immediately following an action, *delayed*, following an action after an interval of time, or *aggregate*, provided after multiple actions are completed. *Aggregate *data visualizations, such as summaries of patient performance across ten reaches, can facilitate overall assessment and comparison of performance across multiple timescales [76].

##### Application

While an action is being performed, motor behaviors may occur along a continuum ranging from application of external information for real-time correction, to reliance on internal models only [77]. Accordingly, feedback can provide information for *online control *(modification of ongoing performance); it can primarily facilitate *feedforward *planning of future movements; or it may provide weighted combinations of both. For example, feedback designed for online control can also be used to correct motor performance in a feedforward manner [17]. Thus feedback components should be constructed that promote the most appropriate usage strategy by the participant. Continuous, visual feedback, for example, often allows for online adjustment of action due to its ability to communicate direction for improvement in real-time and thus encourage explicit information processing. On the other hand, the human brain's strong memory for musical constructs [53,78] promotes the efficacy of audio media as a powerful feedforward tool for movement planning. Although the interactions of music and movement can be complex, the majority of music-assisted movement learning occurs implicitly and subconsciously, similar to the intuitive learning of dance [53,79].

##### An example of composite interactive media within the AMRR system

**Figure 7:**
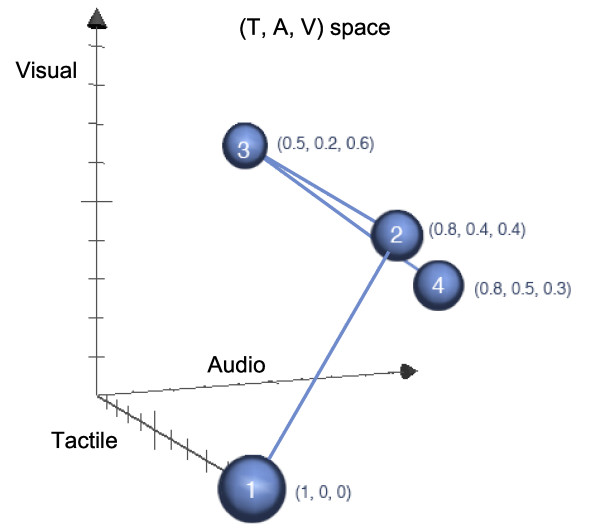
**Three dimensional representation of the sensory modality space during a supported reach to grasp action within the AMRR system**. A three dimensional plot illustrates how the coordinates of the feedback experience change over time. The coordinates of the four points represent (1) when the participant is at rest (hand on table) with no audiovisual feedback, (2) when the participant is prompted by audiovisual feedback prior to the reach but still at rest, (3) when the participant is at the mid point of reaching, and finally (4) when the participant grasps the cone.

The following example of composite feedback is provided from the AMRR system to illustrate how relative weights within the sensory modality space change over time. See additional file 1: AMRR System Demonstration, to view a participant performing a supported (on the table) reach to grasp. The approximate weights of contributing sensory modalities are presented in Figure 7, which depicts 4 points that place the composite feedback in sensory modality space over time. Point 1 represents the feedback experienced prior to the beginning of the reach, when the participant is at rest with no audio or visual media-based feedback provided. Point 2 represents the feedback experienced prior to reach and grasp, when the music begins and the image appears, prompting the participant for action. Point 3 represents feedback experienced approximately midway through the reach, when sensory modalities are more evenly distributed between visual media-based feedback and tangible feedback. Point 4 represents the occurrence of the grasp, when the visual media feedback fades and tactile and audio feedback dominate. Of course these relative distributions of contributing sensory modalities will vary depending upon the participant's sensory impairments or sensitivities. Though these weights are designed to maximize assistance through feedback to the greatest number of participants, it is crucial that they can be adjusted depending upon the participant's needs. For example, an individual who is unable to utilize the tactile modality may rely far more on visual media information, which can be accentuated or reduced as necessary.

Designing hybrid feedback for a mixed reality space requires integration of multi-dimensional features, such as audio and visual media-based feedback, in the presence of the physical environment (supported table, physical cone). Consideration of multidimensional feedback may be extended to the information processing, interaction time structure, and application spaces as well. Finally it should be noted that while the feedback within a mixed reality rehabilitation system is designed to facilitate self-assessment, the presence of the clinician is required to assist the participant throughout therapy. The training clinician of a mixed reality rehabilitation session should provide verbal or physical guidance for the participant whenever necessary if the participant is having difficulty understanding or utilizing the feedback.

#### Form integration and coherence

Compositional form refers to the key components of a structural unit (e.g., key elements within a literary, artistic or musical composition) and the meaning that arises from the interrelationships among these components. Form integration and coherence refer to the use of media composition principles to integrate individual feedback streams into one meaningful and contextually relevant (coherent) form, thus decreasing the amount of cognitive effort required for understanding the multimodal interaction.

When designing complex mediated experiences, *form integration *is facilitated by the appropriate feature selection for constructing individual feedback components and use of appropriate compositional strategies for merging individual feedback components into a unified context. For example, the AMRR system utilizes the visual modality to communicate the most explicit aspects of the media-based task (the goal of the interaction is to successfully complete an image) while the audio modality provides more implicit information often requiring reflection (underlying affect, encouragement and timed progression). This approach draws from well-established compositional techniques of film theory and practice [80]. *Form coherence *refers to the tight coupling and semantic congruence between the content of the media-based feedback and the action that generates the feedback. The goal of the action (e.g., successfully completing a reaching task) and the goal represented in the media (e.g., successfully completing a media-based game) must be analogous. The relative contributions of movement components in achieving the action goal must be reflected in the relationship of the corresponding media elements. In addition to communicating performance, individual feedback streams must also encode the ideal form of their movement components (e.g., a smoothly executed musical feedback progression is generated by an efficient reaching movement) to allow for intuitive communication of error and direction for improvement. Both form integration and form coherence require that the media feedback reflect all key aspects of the entire action, rather than communicating single aspects of movement in isolation.

We propose that form integration and coherence can be achieved by using the architecture of the action representation for structuring the media composition. The action representation identifies the key components and establishes overall interrelationships among components and their roles within the hierarchy of action goal completion. Paralleling this structure in the media composition can create a coherent interactive experience for the participant. For example, in the AMRR system, the goal within the interactive media-based task (the completion of the image and musical progression) directly reflects the completion of the goal of the physical action (accomplishment of the reaching task). Activity-level kinematic parameters are mapped to continuous and prominent audio and visual media that contribute the most to completing the interactive task. Body function-level measures are mapped to discrete visual and sonic indicators that can be toggled on or off. Prominent use of linear visual perspective and smoothly accelerating/decelerating music rhythms in the media encode key invariant elements of the movement (straight trajectory and bell-like speed curve, respectively). The tight coupling between media and action that results from form coherence allows the clinician to intuitively and continuously communicate to the participant the focus and structure of each stage of therapy by selecting which media mappings to enable or intensify.

The coupling between media and action, and the lack of a physical device required for interaction, such as a mouse or a Wii remote, allow for an interactive rehabilitation system that centers attention on recovery of action, with little to no focus on the technology being used. Disruption in the continuous media composition, such as an indication of trajectory error, results from the participant's deviations in his physical reaching movement. This disruption leads to a decoupling of body, activity, and action for the discovery of the error. When using a separate interface (like a Wii remote) connected to non-tightly coupled feedback (as in the Wii tennis game application, in which arm movement in the game environment is not mapped to full arm movement of the user), an error in the feedback will first result in a decoupling of technology and body so the user can improve his learning and management of the technology and its artifacts [19]. This intervening process impedes accurate contemplation of the relationships between physical action, tool and body, even for non-impaired users, and significantly increases the learning challenges faced by impaired users.

#### Adaptive design

A system providing interactive feedback that is appropriate for stroke survivors of various levels of impairment must also be capable of adjusting to different difficulty levels, types of impairment, and types of learning. Furthermore, because the recovery process is dynamic for each participant, the feedback must be adaptable in order to continuously engage, challenge and offer useful performance information to the stroke survivor. The combination of the audio, visual, and tangible (target, table) information that the user experiences while interacting with the system, referred to as the feedback and task environment, must be adaptable along the following dimensions:

*Sensitivity of Media-Based Feedback*: The amount of movement error required to produce observable feedback error must be adaptable to the participant's ability and progress.

*Fading of Media-Based Feedback*: Any number of feedback components must be easily added or subtracted without influencing the effectiveness of other components. Fading allows the partitioning of training into sections that each addresses few movement components so as not to overwhelm the participant.

*Task Type and Sequence*: Multiple types of tasks (e.g., reaching to push a button, or reaching to grasp) must be trainable utilizing similar media mappings across these different tasks to support generalized learning. The order and level of challenge of each task must also be adaptable to the participant's progress.

*Amount of virtual (media-based) and physical (tactile) elements*: Training sequences must range from primarily virtual (the participant controls media-based feedback with his actions) to mixed (the participant interacts with physical objects while assisted by media-based feedback) to purely physical (the participant interacts with a physical object with no augmented feedback). Adaptable environments along a digital-physical continuum help control the level of dissociation from the physical experience (recontextualization) at each point of the training, while connecting learning in the virtual domain to physical action.

## Conclusions and Application within an Adaptive Mixed Reality Rehabilitation System

This paper has integrated phenomenological approaches to interaction and embodied knowledge with rehabilitation practices and theories to achieve a methodology that can support effective adaptive, interactive stroke rehabilitation. A simplified representation of the reach and grasp action space organizes the relationships among key kinematic features of the movement to be trained. These parameters are grouped into overlapping categories reflecting action components at the activity and body function levels. All parameters and categories must be quantified by a reliable and objective method, such as by utilizing motion capture to extract kinematic data. Such quantification allows assessment by the therapist and generation of real-time feedback that promotes self-assessment by the participant. Feedback should intuitively communicate evaluations of the individual kinematic parameters, their interrelationships, and integration as a unified action. This communication can be achieved by using an abstract feedback composition that parallels the form of the action representation and careful selection of appropriate key feedback features (in terms of sensory modality, reception process, interaction time structure, and usage goal). Effective mixed reality rehabilitation systems should be highly adaptable to maintain an appropriate level of challenge and engagement based on the level of impairment and progress.

The principles described here have been applied within an Adaptive Mixed Reality Rehabilitation (AMRR) system for stroke rehabilitation. Results from a current study applying the AMRR system to the upper extremity rehabilitation of stroke survivors have demonstrated improvements across several clinical and functional scales, which support the AMRR system's potential for effective training as a novel adaptive, interactive interface for stroke rehabilitation. The application of principles underlying the AMRR system and a summary of some results from this ongoing study form the basis of our companion paper [81].

## Competing interests

The authors declare that they have no competing interests.

## Authors' contributions

NL, TR, and SW contributed to the concepts and of the paper. NL and SA prepared the manuscript. TR and SW provided editing and consultation. All authors read and approved the final manuscript.

## Supplementary Material

Additional file 1 **AddFile1_AMRRSystemDemonstration.mov**. QuickTime movie. Depicts a participant interacting with the system while performing a supported reach. Click here for file
